# Lipid exposure leads to metabolic dysfunction in fetal sheep cardiomyocytes

**DOI:** 10.14814/phy2.70386

**Published:** 2025-05-26

**Authors:** Natasha N. Chattergoon, Karthikeyan Bose, Samantha Louey, Sonnet S. Jonker

**Affiliations:** ^1^ Center for Developmental Health, Knight Cardiovascular Institute Oregon Health & Science University Portland Oregon USA

**Keywords:** cardiomyocyte, fetus, metabolism, parenteral nutrition

## Abstract

Fetal circulating lipids are low but rise precipitously following birth. It is unknown how prematurely elevated lipids affect the fetal heart, which primarily uses carbohydrates for energy. Fetal sheep were surgically instrumented and received Intralipid 20® or Lactated Ringer's Solution intravenously. After 8 days, myocardial biopsies were taken, and cardiomyocytes were dispersed. Lipid uptake was assessed by labeled saturated long‐chain fatty acids (LCFA) and very long‐chain fatty acids (VLCFA) incorporation. Maximal oxygen consumption rates (OCR) were measured. Gene and protein expression levels were measured by quantitative PCR and Western blotting. Intralipid treatment increased LCFA (*p* < 0.001) and VLCFA (*p* < 0.001) lipid droplet number, and LCFA (males *p* = 0.002) and VLCFA (*p* = 0.018) droplet size. Fetal Intralipid treatment reduced maximal OCR in basal media (*p* = 0.005). Palmitic acid decreased maximal OCR regardless of fetal treatment or length of in vitro exposure (*p* = 0.006). Fetal Intralipid upregulated genes included CD36 (*p* = 0.001), CPT1A (*p* < 0.001), CPT1B (*p* < 0.001), VLCAD (*p* < 0.001), and PDK4 (*p* < 0.001), with no differences in protein expression. There were no effects on ER stress, apoptosis, or autophagy markers. Extended elevated lipid levels in the fetus increased lipid uptake and may have shifted substrate preference towards lipids, but all lipid exposure depressed fetal cardiac metabolism. Prematurely elevated lipids mature but suppress oxidative metabolism.

## 1 INTRODUCTION

In the fetus, circulating fatty acid levels are very low, and there is very little uptake or oxidation of fatty acids by the heart (Bartelds et al., 1998, 2000; Lopaschuk & Jaswal, 2010). There is progressive maturation of the cardiac capacity for fatty acid oxidation in the fetal and newborn period (Bartelds et al., 1998, 2000; Lopaschuk & Jaswal, 2010). Exposure to fatty acids may contribute to the maturation of the metabolic machinery of the newborn heart, as newborn hearts do not oxidize as much fatty acid as juvenile hearts, despite similar circulating fatty acid levels (Bartelds et al., 1998; Lopaschuk & Jaswal, 2010).

Preterm newborns are born with immature organ systems and require support and care different from that of a healthy term newborn. In order to support optimal brain growth, it is necessary to supply preterm infants with high‐lipid nutrition (Duttaroy & Basak, 2021; Steiner, 2019). It is recommended that parenteral nutrition (PN) be initiated immediately after birth in infants born before 37 weeks (Robinson et al., 2023). PN is weaned as soon as the neonate gains the capacity for enteral feeding, although the timing of this is highly variable and ranges from a day to months (Delgado Paramo et al., 2024; Robinson et al., 2023). PN lipids sometimes cause lipotoxic stress in the preterm heart, leading to long‐term consequences including a large aortic root, aortic stiffness, and reduced left ventricular peak systolic circumferential strain (Hamayun et al., 2021; Lewandowski et al., 2011). It is unknown, however, if extended exposure to high lipid levels accelerates maturation or causes dysregulation of the metabolic machinery of the preterm heart.

We infused fetal sheep at a gestational age equivalent to 36 weeks with Intralipid 20® at a clinically indicated dose and then studied their hearts in order to understand how exposure to high lipid levels altered cardiomyocyte function. We selected 8 days of exposure to be longer than the 7‐day minimum exposure period required in many clinical PN outcome studies (Kapoor et al., 2019). We hypothesized that Intralipid exposure would increase the capacity of fetal cardiomyocytes to take up and oxidize fatty acids, but that the exposure would also cause endoplasmic reticulum (ER) stress and activate protective pathways including autophagy or apoptosis.

## 2 METHODS

### 2.1 Animals

All animal experiments were approved by the Institutional Animal Care and Use Committee (#IP0007) and conducted at Oregon Health & Science University. Details of the animal experiment methods, fetal blood pressures, fetal arterial blood gases and contents, and pH have been published (Piccolo et al., 2024). Briefly, ewes in good body condition carrying timed‐bred twin fetuses were obtained from a local supplier (Agna LLC, Salem, Oregon). Fetal sheep were surgically catheterized at 119 ± 1 days of gestational age (dGA) to measure intravascular pressures, to take blood samples, and to permit intravenous infusion. Immediately following surgery, pain was controlled by subcutaneous Buprenex (0.3 mg buprenorphine HCl; Covetrus, Dublin, OH) and sustained release buprenorphine (0.05 mg kg^−1^, Wedgewood Pharmacy, Swedesboro, NJ), and the recovery period was 6 ± 1 days. Following recovery, fetuses (four females and seven males) were infused via an indwelling jugular vein catheter with Intralipid 20® (Frensenius Kabi, Lake Zurich, IL, Cat. No. 831800311) according to the manufacturer's recommendations for premature human infants (Duttaroy & Basak, 2021). They received an initial dose of 0.5–1.0 g kg^−1^ d^−1^, increased by 0.5–1.0 g kg^−1^ d^−1^ to a maximum of 3 g kg^−1^ d^−1^. Actual dose achieved was calculated from necropsy weight: initial infusion rate (day 0) was 0.7 ± 0.1 g kg^−1^ d^−1^ (0.142 ± 0.03 mL kg^−1^ h^−1^), by day 4 the rate was 2.6 ± 0.5 g kg^−1^ d^−1^, (0.540 ± 0.10 mL kg^−1^ h^−1^), and on the final day the rate was 2.8 ± 0.5 g kg^−1^ d^−1^ (0.595 ± 0.11 mL kg^−1^ h^−1^; daily rate values missing for one subject). Control fetuses (seven females and four males) received Lactated Ringer's Solution at an equal volume. Gestational age on experimental day 0 was 125 ± 1 dGA (days of gestational age; term is 147 dGA) and 133 ± 1 dGA at the conclusion of the experiment (day 8).

Ewes were humanely euthanized with an intravenous overdose of a commercial sodium pentobarbital solution. Fetuses were given a bolus dose of 10 mL heparin (Frensenius Kabi, NDC 63323‐540‐15) and 10 mL saturated KCl (MilliporeSigma, St Louis, MO, Cat. No. P9333) via the umbilical vein to arrest the heart in diastole. Fetal weight and sex were recorded, and then the heart was dissected. A small left ventricular midventricular biopsy was frozen in liquid nitrogen for molecular analysis. The heart was then enzymatically dissociated to obtain isolated cardiomyocytes for live imaging studies (Chattergoon et al., 2023; Jonker et al., 2007). Briefly, a wet seal was made between a cannula and the ascending aorta, which was then perfused with Tyrode solution (all chemicals from MilliporeSigma: 140 mM NaCl [Cat. No. S9625], 5 mM KCl [Cat. No. P9333], 1 mM MgCl_2_ [Cat. No. M8266], 10 mM dextrose [Cat. No. G8270], and 10 mM HEPES [Cat. No. H4034], pH adjusted to 7.35 with NaOH [Cat. No. S5881]) until the myocardium blanched. The digestion solution followed for ~5 min: Tyrode solution with 160 U/mL Worthington type II collagenase (Worthington Biochemicals, Lakewood, NJ, Cat. No. CLS‐2) and 0.78 U/mL protease type XIV (MilliporeSigma, Cat. No. P5147). Calcium‐free Kraftbrühe buffer (all chemicals from MilliporeSigma: 74 mM L‐glutamic acid [Cat. No. G1501], 30 mM KCl [Cat. No. P9333], 30 mM KH_2_PO_4_ [Cat. No. P5655], 20 mM taurine [Cat. No. T8691], 3 mM MgSO_4_ [Cat. No. M7506], 0.5 mM EGTA [Cat. No. E3889], 10 mM HEPES [Cat. No. H4034], and 10 mM dextrose [Cat. No. G8270], pH adjusted to 7.37 with KOH [Cat. No. P1767]) was used to rinse out the collagenase. All perfusion solutions were preheated to 39°C and bubbled with a 95% O_2_–5% CO_2_ gas mixture. The ventricular walls were individually dissected free, scored with scissors, and gently agitated in Kraftbrühe (KB) buffer. The myocyte slurry was set aside at room temperature for ~30 min.

### 2.2 Live cell imaging of fatty acid uptake

The incorporation of exogenous saturated long‐chain fatty acids (LCFA; 18‐carbon) and very long‐chain fatty acids (VLCFA; 22‐carbon) into lipid droplets in freshly isolated fetal cardiomyocytes was measured using an established lab protocol (Chattergoon et al., 2023; Drake et al., 2022). Solutions of fatty acids attached to intensely fluorescent BODIPY™ (4,4‐difluoro‐3a,4a‐diaza‐s‐indacene) in DMSO were diluted to 2.5 mM KB solution (1:250; supplemented [all chemicals from MilliporeSigma] with 2 mM glutamine [Cat. No. G8540], 200 μM sodium pyruvate [Cat. No. P5280], 2 mM lactate [Cat. No. L7022], 1 mM glucose [Cat. No. G8270], and 500 μM carnitine [Cat. No. C0158]) with 0.1% weight per volume fatty acid free bovine serum albumin (BSA; Thermo Fisher, Cat. No. BP9704100). Freshly isolated left ventricular cardiomyocytes were incubated in 8‐well μ‐slides (Ibidi Inc., Fitchburg, WI, Cat. No. 80821) for 60 min (39°C, 5% CO2) in KB supplemented with these labeled fatty acids: BODIPY™ C12 (2 μM; Invitrogen, purchased from ThermoFisher Scientific, Cat. No. D3822) or BODIPY™ C16 (2 μM; Invitrogen, purchased from ThermoFisher Scientific, Cat. No. D3821). After 60 min, z‐stack images were collected using the 63× oil lens on a Zeiss 880 LSM with Airyscan software with a 488 laser (intensity 0.6, gain 825, digital gain 1.0). For each animal, 20–35 cells were measured over an average of seven frames (range of 3–12). A total of 90–130 slices (0.2 μm thick) were acquired per frame (total z‐axis thickness of 18–26 μm was greater than any cardiomyocyte diameter). Z‐stacks were processed in Airyscan and maximum intensity projections performed in ZEN Black Software (Carl Zeiss Inc., Thornwood, NY, USA). Fiji software was used to analyze all images obtained as described (Drake et al., 2022). Images were processed using Enhance Local Contrast (CLAHE: blocksize = 9, histogram = 256, maximum = 4), despeckled, and background subtracted (rolling = 5). Lipid droplet particles were analyzed by filtering for the following parameters: circularity (0.8–1), size from 0.0314 μm (minimal detectable size for the LSM880) to 3 μm (exceeds the maximum non adipocyte lipid droplet size) (Wang et al., 2013). Images were analyzed using AutoThreshold (Intermodes dark function) with conversion to a mask for lipid droplets and entire cells. Masks were manually verified to ensure the parameters did not accidentally exclude viable or include nonviable cells. Lipid droplet number was referenced to total cardiomyocyte area to calculate lipid droplet density. Cardiac dissociation did not produce viable cells in one fetus; thus, the number of males in the Intralipid group was reduced to 6. After data analysis, to improve the image quality for visual presentation in the figure, the output of the green channel was adjusted without changing the ratio of intensities among cardiomyocytes (the input: output linear curve for each image, excluding the scale bar, was shifted from 1:1 to 1:2 without changing the intercept).

### 2.3 Seahorse analysis of maximal cellular respiration

Culture of isolated fetal sheep cardiomyocytes, treatment with palmitic acid, and quantitative analysis of cellular respiration were carried out as previously described (Chattergoon et al., 2023). Freshly isolated left ventricular cardiomyocytes from Control and Intralipid fetuses were preplated in 10% serum media (Gibco DMEM, Cat. No. 11885092, Gibco Fetal Bovine Serum, Heat Inactivated, Cat. No. 10082147) in T‐75 flasks (7–12 million flask^−1^) until ~70% confluent (typically 2–3 days) (Chattergoon et al., 2007, 2023; Sundgren et al., 2003). Cells received fresh serum media for 24 h and then were released from the flask with trypsin (0.25% trypsin–EDTA, Gibco Cat. No. 25200114). Cells were resuspended in cryoprotectant (10% DMSO, MilliporeSigma, Cat. No. D2650, 90% FBS; 1–10 million cells ml^−1^) and gradually cooled (−1°C min^−1^) in a Mr. Frosty Freezing Container (Thermo Fisher, Cat. No. 5100–0001) in a −80°C freezer for 24–48 h and then transferred to liquid nitrogen storage. To retrieve cells for experiments, cryotubes were warmed for 30 s in a 39°C water bath and the thawed cells added to warmed serum‐supplemented media (Chattergoon et al., 2014; O'Tierney et al., 2010). Trypan blue (MilliporeSigma, Cat. No. T8154) exclusion was used to determine cell viability and number. Cardiomyocytes at passage 1 were seeded at 20,000 cells per well^−1^ in serum‐supplemented media for 24 h before being changed to serum‐free (SF) media for 24 h. In one set of experiments, cells from Control and Intralipid fetuses were assessed after establishment in culture, with no treatments. In a separate set of experiments, cardiomyocytes were treated with palmitic acid (PA) (50, 100, 200, or 500 μM, Thermo Fisher, Cat. No. 129702500) with fatty acid‐free bovine serum albumin (BSA; 6:1 ratio; Fisher BioReagents, Cat. No. BP9704100) and 50 μM carnitine (MilliporeSigma, Cat. No. C0158) supplementation, or the BSA vehicle at an equal concentration, acutely (30 min), 24 h, or 48 h prior to assessment. To prepare cells for the assay, they were washed twice and left to equilibrate for 1 h in room air at 39°C in non‐buffered base medium (Agilent Technologies Inc.) supplemented with 2 mM glutamine (ThermoFisher, Cat. No. 25030081), 200 μM sodium pyruvate (MilliporeSigma, Cat. No. P5280), 2% FBS, 1 mM glucose, and 2 mM l‐lactate. Oxygen consumption rates (OCR) were measured in a mitochondrial stress test with the XFe96 Seahorse Extracellular Flux Analyzer (Agilent Technologies Inc., Santa Clara, CA, USA). Following the calibration and equilibration period, 3 min measurements were taken three times alternating with 3 min of mixing at (1) basal conditions (reflective of basal respiration); (2) after introduction of the ATP synthase inhibitor oligomycin, 1.5 μM (reflective of proton leak; MilliporeSigma, Cat. No. 75351); (3) after introduction of the mitochondrial uncoupling agent carbonyl cyanide‐p‐trifluoromethoxyphenylhydrazone (FCCP; 2.7 μM; reflective of maximal respiration; MilliporeSigma, Cat. No. C2920); and (4) after introduction of the complex I and cytochrome c reductase inhibitors rotenone‐antimycin A, 1 μM (reflective of non‐mitochondrial respiration; MilliporeSigma, Cat. No. R8875). Too few cells were collected from two fetuses (one control male and one experimental male) to assess all timepoints.

### 2.4 Quantitative PCR analysis of mRNA expression levels

mRNA was extracted, first‐strand cDNA was synthesized, and quantitative PCR was performed as previously described (Chattergoon et al., 2023). Left ventricular myocardium was homogenized in TRIzol with a steel bead in a TissueLyser LT (Qiagen, Germantown, MD). The TRIzol protocol (Invitrogen, Carlsbad, CA, USA) was used to extract RNA. The RNeasy protocol (Qiagen, Cat. No. 74004) was used to purify samples. Applied Biosystems™ High‐Capacity cDNA Reverse Transcription Kit with RNase inhibitor (Thermo Fisher Cat. No. 4374966) was used to synthesize first‐strand cDNA. Table 1 shows primers (Eurofins Genomics, Louisville, KY) for genes studied (MX3005P qPCR system, Agilent Technologies Inc.). Optimal cycling conditions were experimentally determined for each primer pair, and the size of each PCR product was verified on a 1% agarose gel. In subsequent runs, the melting curve analysis was used to verify a single product of expected size that melted at the expected temperature. Fluorescence measurements during the PCR reactions were monitored to determine the amount of double‐stranded DNA present. For each unknown sample, relative amounts of transcripts were calculated by the standard curve method and referenced to RPL37A.

**TABLE 1:** Primers used for quantitative PCR.

Full name	Forward sequence	Reverse sequence	Accession #	Product (bp)
Fatty acid transporters				
CD36 molecule (CD36)	CTGGTGGAAATGGTCTTGCT	ATGTGCTGCTGCTTATGGGT	XM_027968558.1	185
Carnitine palmitoyltransferase 1A (CPT1A)	GAGACACCAACCCGAAGATC	GTCTCTGTTCTGCCCTCTCG	NM_001009414.1	295
Carnitine palmitoyltransferase 1B (CPT1B)	GGATGTTTGAGATGCACGGC	GCCAGCGTCTCCATTCGATA	XM_027965744.1	214
Signaling				
Peroxisome proliferator activated receptor alpha (PPARA)	ATCGAGTGTAGGATCTGCGG	GAATCGCGTTATGGGACATC	XM_015095065.1	217
Very low density lipoprotein receptor (VLDLR)	GCATTGCTCACATTTCACGC	TGGTTGTACGCTCTGGACTT	XM_060409407.1	165
Esterification and lipid droplet formation				
Diacylglycerol O‐acyltransferase (DGAT)	AGACACTTCTACAAGCCCATGCTC	AGTGCACTGACCTCATGGAAGA	XM_027972747.1	264
Glycerol‐3‐phosphate acyltransferase (GPAT)	GAAGTGGCTGGTGAGTTAAACCCT	CAGTCTGATCATTGCCGGTGAAAC	XM_012102930.2	132
Phospholipid phosphatase 1 (PLPP1; also known as phosphatidic acid phosphatase (PAP))	AGAATGAAGGGAGACTGGGCAAGA	GCAACCAGAGCTCCTTGAATGAGT	XM_004016984.4	152
Βeta‐oxidation				
Hydroxyacyl‐CoA dehydrogenase (HADH)	AGAAAACCCCAAGGGTGCTGAT	GCCTCTTGAACAGCTCGTTCTT	XM_004009637.4	152
Long chain acyl‐CoA dehydrogenase (LCAD; also known as ACADL)	TGAAAGCCGCATTGCCATTGAG	ACTTGGATGGCCCGGTCAATAA	XM_004003336.4	159
Very long chain acyl‐CoA dehydrogenase (VLCAD; also known as ACADVL)	AAGATCCCTGAGTGAAGGCCA	TAGAACCAGGATGGGCAGAAA	XM_004012636.3	291
Metabolism and tricarboxylic acid cycle				
Isocitrate dehydrogenase, NADP(+) (IDH)	CTGTGTTTGAGACGGCTACAAGGA	CGTAGCTGTGGGATTGGCAATGTT	XM_027963525.1	74
Pyruvate dehydrogenase kinase 4 (PDK4)	CCTGTGATGGATAATTCCCG	TTGGTTCCTTGCTTGGGATA	XM_004007738.3	259
Endoplasmic reticulum stress				
Activating transcription factor 6 (ATF6)	AGTTCTGCCCTCTCCTCAAC	GGGACTGACAAGCTGACTCT	XM_012185020.3	230
DNA damage inducible transcript 3 (DDIT; also known as C/EBP homologous protein (CHOP))	TGAGTCATTGCCGTTCTCCT	AGGGTCAAGAGTGGTGAAGG	XM_004006542.5	163
G‐protein coupled receptor 78 (GRP78; also known as Heat shock protein family A (Hsp70) member (HSPA5))	AGGACAAGAAGGAGGACGTG	TCAGGATTGGAGGTGAGCTG	XM_060409407.1	225
Autophagy				
Beclin 1 (BECN1)	ACTGGACACGAGCTTCAAGA	GATGCCTCCCCAATCAGAGT	XM_060395652.1	190
Reference				
RPL37A	ACCAAGAAGGTCGGAATCGT	GGCACCACCAGCTACTGTTT	XM_012166909.1	192

### 2.5 Western blot analysis of protein expression levels

Western blot was carried out as previously described (Chattergoon et al., 2023). Frozen left ventricular myocardium was homogenized using a steel bead in the TissueLyser LT (Qiagen) in Upstate RIPA lysis solution (MilliporeSigma, Burlington, VT, Cat. No. 20‐188) with Roche cOmplete™ Mini Protease Inhibitor Cocktail (MilliporeSigma, Cat. No. 11836153001). Extracted protein was quantified by BCA assay (Pierce, Rockford, IL, USA). Protein samples (10 μg) were separated by SDS‐PAGE on a 4%–20% gradient Novex™ Value™ Tris‐glycine gel (ThermoFisher Scientific) and transferred to a Whatman® Optitran® BA‐S 83 nitrocellulose membrane (Millipore Sigma). Membranes were blocked with 5% milk in 1X Tris buffered saline with 0.01% Tween 20 (TBS‐T) buffer for 1 h at room temperature. Membranes were incubated with primary antibodies (1:1000) overnight at 4°C in 1X TBS‐T buffer with 4% BSA (Table 2). Large volumes of TBS‐T were used to wash membranes three times for 10 min each before exposure to the horseradish peroxidase‐conjugated secondary antibody (1:5000) in TBS‐T with 5% nonfat milk for 1 h at room temperature. Membranes were then washed as above. Antibody binding was detected using chemiluminescence (SuperSignal, Pierce, IL, USA), developed on CL‐XPosure TM film (Thermo Fisher Scientific), and quantified from a digitized image using NIH ImageJ (Version 1.53e) (Schneider et al., 2012). Signal density of proteins of interest was normalized to α‐tubulin for each respective sample. After densitometry of raw images, representative images were prepared by juxtaposition with the ladder image of the same blot and auto white balance to adjust contrast for the entire image.

**TABLE 2:** Antibodies used for Western Blot.

Protein target	Molecular weight	Vendor	Catalog number	RRID
CD36	75–85 kDa	Abcam	ab133625	AB_2716564
CPT1A	88 kDa	Cell Signaling Technology	12252	AB_2797857
CPT1B	88 kDa	Abcam	ab104662	AB_10712608
PPARA	42–45 kDa	Abcam	ab24509	AB_448110
Total EIF2A	38 kDa	Cell Signaling Technology	5324	AB_10692650
Phospho‐EIF2A	38 kDa	Cell Signaling Technology	3398	AB_2096481
Cleaved caspase 3	17, 19 kDa	Cell Signaling Technology	9664	AB_2070042
Tubulin alpha 1B chain (TUBA1B)	52 kDa	Cell Signaling Technology	2125	AB_2619646
Anti‐rabbit IgG		Cell Signaling Technology	7074	AB_2099233

### 2.6 Statistics

Dichotomous variables were assessed by Fisher's exact test. This analysis was carried out in GraphPad Prism (v.10.1.0). Normality of continuous data was assessed by Shapiro–Wilk's test (*p* > 0.05). Homogeneity of variances was assessed by Levene's test for equality of variances. Outliers were assessed as being 1.5 box‐lengths or more from the edge of the box in a boxplot. For comparison by in vivo treatment and sex, data were assessed by 2‐way ANOVA; multiple comparisons with the Bonferroni correction were performed if indicated. Seahorse data were assessed by unpaired, 2‐tailed *t*‐test or by mixed measures 3‐way ANOVA (factors: in vivo treatment, in vitro treatment, in vitro treatment duration) using the Greenhouse–Geisser correction for sphericity. Statistical analyses were carried out in SPSS (v.29.0.0.0). Significance was defined at *α* = 0.05.

## 3 RESULTS

### 3.1 Lipid uptake and droplet formation

**FIGURE 1:**
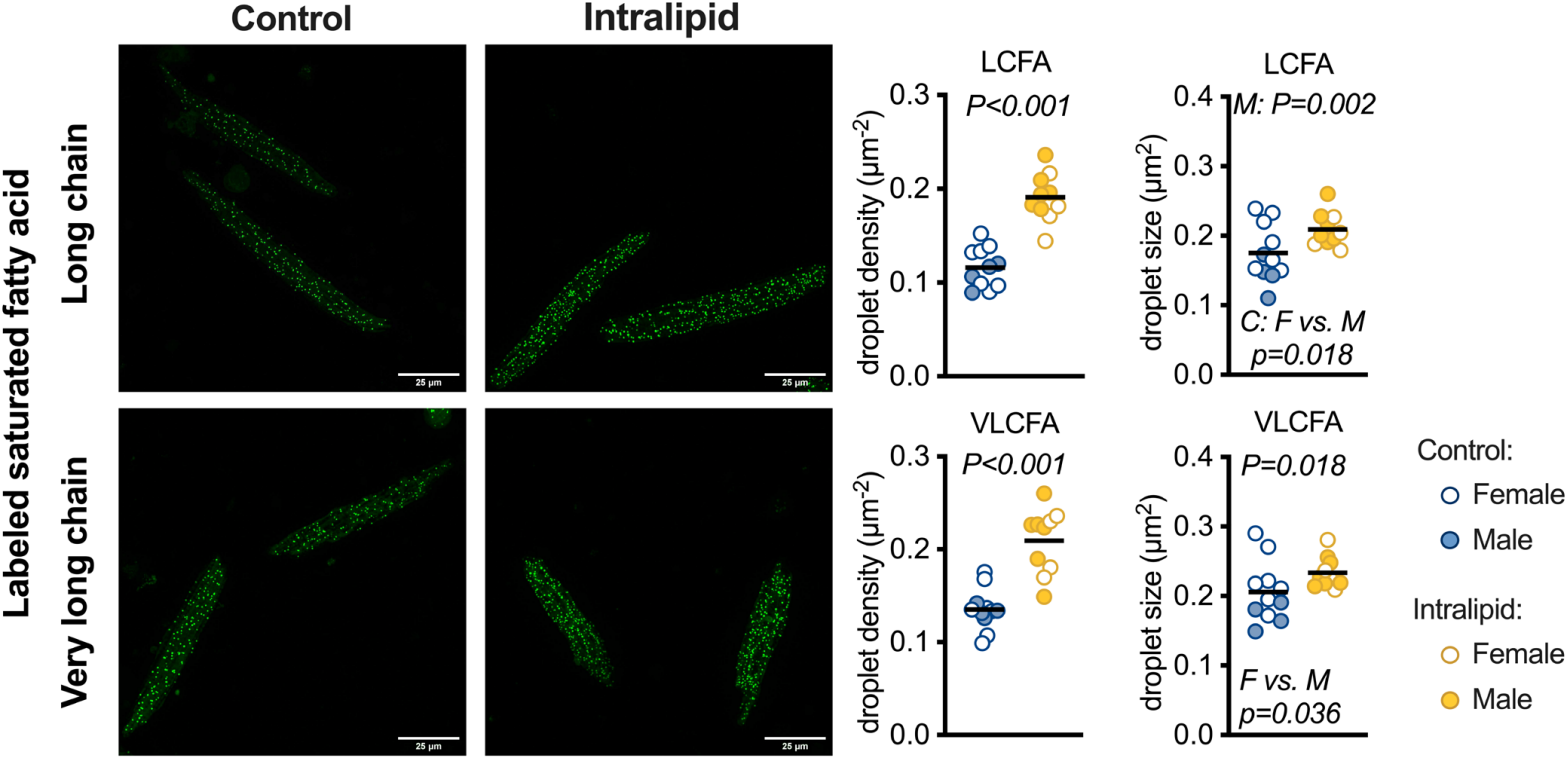
Lipid uptake and droplet formation in isolated cardiomyocytes following in vivo Intralipid administration in fetal sheep. Left panels: Representative images of BODIPY™‐labeled fatty acid incorporation into cellular lipid droplets (green; magnification 630×). Right panels: Summarized data of labeled long‐chain fatty acid (LCFA; upper panels) or very long‐chain fatty acid (VLCFA; lower panels) lipid droplet number per cell area and droplet size in left ventricular cardiomyocytes from Intralipid‐infused fetuses compared to vehicle‐infused fetuses. Scale bars = 25 μm. Raw data with mean. Number for Control (C) female (F) = 7, male (M) = 4; Intralipid female = 4, male = 6. Data were assessed by 2‐way ANOVA. Significance determined at *α* = 0.05 and shown for treatment main effects unless otherwise noted. Simple main effects tested following significant interaction using Bonferroni correction for multiple comparisons; family‐wise *α* = 0.025.

Exposure to 8 days of in vivo Intralipid increased ex vivo fetal cardiomyocyte lipid uptake and droplet formation (Figure 1 and Table 3). Numerical density of LCFA droplets was 65% greater in Intralipid‐treated fetal cardiomyocytes versus Controls (*p* < 0.001). There was an interaction between treatment and fetal sex for LCFA lipid droplet size (*p* = 0.027). LCFA droplets were 50% larger in male fetuses treated with Intralipid versus Controls (*p* = 0.002). Further, within the Control group, lipid droplets from female fetuses were 34% larger than those from male fetuses (*p* = 0.018).

**TABLE 3:** *p* Values from two‐way analyses of variance of in vivo treatment and sex.

	2‐way interaction	Main effects		Subgroup data characteristics		
		Test only if 2‐way interaction is NS		Normal distribution	Homogeneity of variances	Outliers
	In vivo treatment × sex	In vivo treatment	Sex			
Lipid droplet analysis						
LCFA droplet numeric density	0.126	**<0.001**	0.672	4/4	Yes	0
LCFA droplet size	**0.027** a	**–**	–	4/4	Yes	0
VLCFA droplet numeric density	0.672	**<0.001**	0.847	4/4	Yes	0
VLCFA droplet size	0.108	**0.018**	**0.036**	4/4	Yes	0
Fatty acid transporters mRNA						
CD36	0.648	**0.001**	0.167	4/4	Yes	1 d
CPT1A	0.757	**<0.001**	0.990	4/4	No c	1 d
CPT1B	0.994	**<0.001**	0.119	4/4	Yes	1 d
Fatty acid transporters protein						
CD36	0.949	0.754	0.321	2/4 b	Yes	1 d
CPT1A	0.612	0.372	0.774	2/4 b	Yes	1 d
CPT1B	0.639	0.481	0.532	3/4 b	Yes	1 d
Esterification and lipid droplet formation mRNA						
DGAT	0.323	0.119	0.389	4/4	Yes	1 d
GPAT	0.630	0.244	0.747	1/4 b	Yes	2 d
PLPP1	0.188	0.119	0.725	4/4	No c	0
Βeta‐oxidation mRNA						
HADH	0.372	0.293	0.802	4/4	Yes	2 d
LCAD	0.397	0.196	0.346	4/4	Yes	1 d
VLCAD	0.300	**<0.001**	0.880	3/4 b	Yes	2 d
Metabolism and tricarboxylic acid cycle mRNA						
IDH	0.982	0.971	0.377	3/4 b	Yes	2 d
PDK4	0.645	**<0.001**	0.652	3/4 b	No c	3 d
Signaling mRNA						
PPARA	0.336	**0.030**	0.914	4/4	Yes	2 d
VLDLR	0.523	0.268	0.055	4/4	No c	0
Signaling protein						
PPARA	0.937	0.303	0.606	3/4 b	Yes	1 d
Endoplasmic reticulum stress mRNA						
ATF6	0.403	0.244	0.247	4/4	Yes	0
DDIT3	0.276	0.475	0.340	4/4	Yes	0
HSPA5	0.819	0.571	0.337	4/4	Yes	0
Endoplasmic reticulum stress protein						
Phospho‐EIF2A	0.594	0.911	0.282	3/4 b	Yes	1 d
Apoptosis protein						
Cleaved caspase 3	0.126	0.812	**0.048**	3/4 b	Yes	1 d
Autophagy mRNA						
Beclin1	0.617	0.222	0.299	4/4	Yes	1 d

*Note*: Experimental genes were normalized to RPL37A. Experimental proteins were referenced to TUBA1B except phospho‐EIF2A was referenced to total EIF2A. Number for Control female = 7, male = 4; Intralipid female = 4, male = 7. Two‐way ANOVA. If indicated, multiple comparisons made with Bonferroni correction. Not significantly different (NS). *p* Values for simple main effects following significant interaction using Bonferroni correction for multiple comparisons and adjusting to family‐wise *α* = 0.025: (a) Control versus Intralipid Female = 0.735 (NS), Male = 0.002; Female versus Male Control = 0.018, Intralipid = 0.426 (NS). (b) Only indicated number of subgroups were normally distributed. Although ANOVAs are fairly robust to deviations from normality, interpret results with caution. (c) Although ANOVAs are fairly robust to heterogeneity of variance, interpret results with caution. (d) Outliers determined to be biologically relevant and included in analysis. SPSS provides significant digits down to 0.001 and *p* <0.001 as bold.

Intralipid treatment also affected uptake and droplet formation for VLCFA (Figure 1 and Table 3), although there were no interactions between treatment and fetal sex. VLCFA numerical density was 55% greater in Intralipid‐treated fetuses than in Controls (*p* < 0.001). Droplet size was 13% greater with Intralipid treatment (*p* = 0.018), and 11% greater in females versus males (*p* = 0.036).

### 3.2 Cellular respiration

**FIGURE 2:**
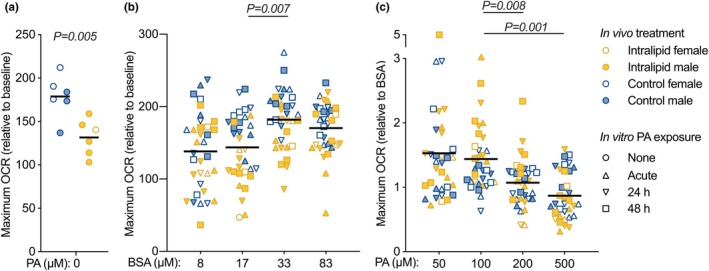
Maximal cellular respiration in cardiomyocytes provided palmitic acid following in vivo Intralipid administration in fetal sheep. (a) Oxygen consumption rates (OCR) were measured in cultured fetal cardiomyocytes from Intralipid or vehicle‐treated fetal sheep exposed only to standard cardiomyocyte media without palmitic acid using the Seahorse Extracellular Flux Analyzer. Data are expressed normalized to baseline OCR. Data were assessed by unpaired *t*‐test. (b) Palmitic acid must be conjugated at a 6:1 molar ratio with bovine serum albumin (BSA) for in vitro treatment. To assess the effects of this vehicle, cardiomyocytes were given fatty acid‐free BSA at a range of doses either acutely during the metabolic analysis, or for 24 h or 48 h prior to measurement. (c) Cardiomyocytes from Intralipid or vehicle‐treated fetal sheep were given palmitic acid (PA) at a range of doses either acutely at the time of measurement, for 24 h, or for 48 h prior to measurement. Data are expressed normalized to the appropriate dose of the bovine serum albumin vehicle. Data for (b) and (c) were assessed by mixed measures 3‐way ANOVA (four levels of repeated measures for in vitro treatment, three levels for in vitro treatment duration) with the Greenhouse–Geisser correction for sphericity (*α* = 0.05; details in Table 4). As there were no significant interactions analysis by in vivo treatment or duration of in vitro PA exposure, data are grouped only by in vitro treatment in the graph; *p* values for multiple comparisons using Bonferroni correction following significant main effects are shown. Numbers for Control = 6 (female = 3, male = 3), Intralipid = 6 (female = 1, male = 5), except for acute in vitro treatment Control = 5 (female = 3, male = 2), Intralipid = 5 (female *n* = 1, male = 4). All data raw with mean.

Exposure to 8 days of in vivo Intralipid reduced maximal oxygen consumption rate (OCR) in a medium without lipids by 24% (*p* = 0.005; Figure 2a).

Cardiomyocytes treated in vitro with palmitic acid (PA) could not be compared to untreated cells due to the effect of the vehicle BSA on OCR (Figure 2b and Table 4). Increasing levels of PA were associated with decreased maximal OCR in a lipid‐containing medium, with no difference by length of exposure or in vivo treatment (*p* = 0.006; Figure 2c; Table 4). Cells treated with 200 μM PA had a 26% lower maximal OCR in a lipid‐containing medium than cells treated with 100 μM PA (*p* = 0.008), while cells treated with 500 μM PA had a 40% lower maximal OCR (*p* = 0.001).

**TABLE 4:** *p* Values from three‐way mixed analyses of variance of in vivo treatment, in vitro treatment, and in vitro treatment duration.

					Main effects					
	3‐way interaction	2‐way interactions			Test only if 2‐ and 3‐way interactions are not significant					
	In vivo treatment × in vitro treatment × in vitro duration	Test only if 3‐way interaction is not significant						Subgroup characteristics		
		In vivo treatment × in vitro treatment	In vivo treatment × in vitro duration	In vitro treatment × in vitro duration	In vivo treatment	In vitro treatment	In vitro duration	Normal distribution	Homogeneity of variances	Outliers
In vitro BSA effect on maximal OCR	0.626	0.311	0.616	0.092	**0.013**	**0.006** a	0.254	22/24 c	11/12 d	10 e
In vitro PA effect on maximal OCR (referenced to BSA)	0.795	0.710	0.124	0.517	0.337	**0.006** b	0.092	22/24 c	12/12	12 e

*Note*: Number for Control = 6 (female = 3, male = 3), Intralipid = 6 (female = 1, male = 5) except for acute treatment Control = 5 (female = 3, male = 2), Intralipid = 5 (female = 1, male = 4); insufficient numbers for comparison by sex. Mixed measures three‐way ANOVA (four levels of repeated measures for in vitro treatment, three levels for in vitro treatment duration) with the Greenhouse–Geisser correction for sphericity. *p* values for multiple comparisons using Bonferroni correction following significant main effects: (a) BSA 17 μM (equivalent to BSA in 100 μM PA well) versus 33 μM (equivalent to BSA in 200 μM PA well) = 0.007. (b) PA 100 μM versus 200 μM = 0.008, versus 500 μM = 0.001. (c) Only indicated proportion of subgroups were normally distributed. Although ANOVAs are fairly robust to deviations from normality, interpret results with caution. (d) Only indicated proportion of subgroups had homogeneity of variances. Although ANOVAs are fairly robust to heterogeneity of variance, interpret results with caution. (e) Number of outliers found and determined to be biologically relevant and included in analysis. SPSS provides significant digits down to 0.001 and *p* < 0.001 as bold.

Abbreviations: BSA, bovine serum albumin; OCR, oxygen consumption ratio; PA, palmitic acid.

### 3.3 Gene and protein expression

**FIGURE 3:**
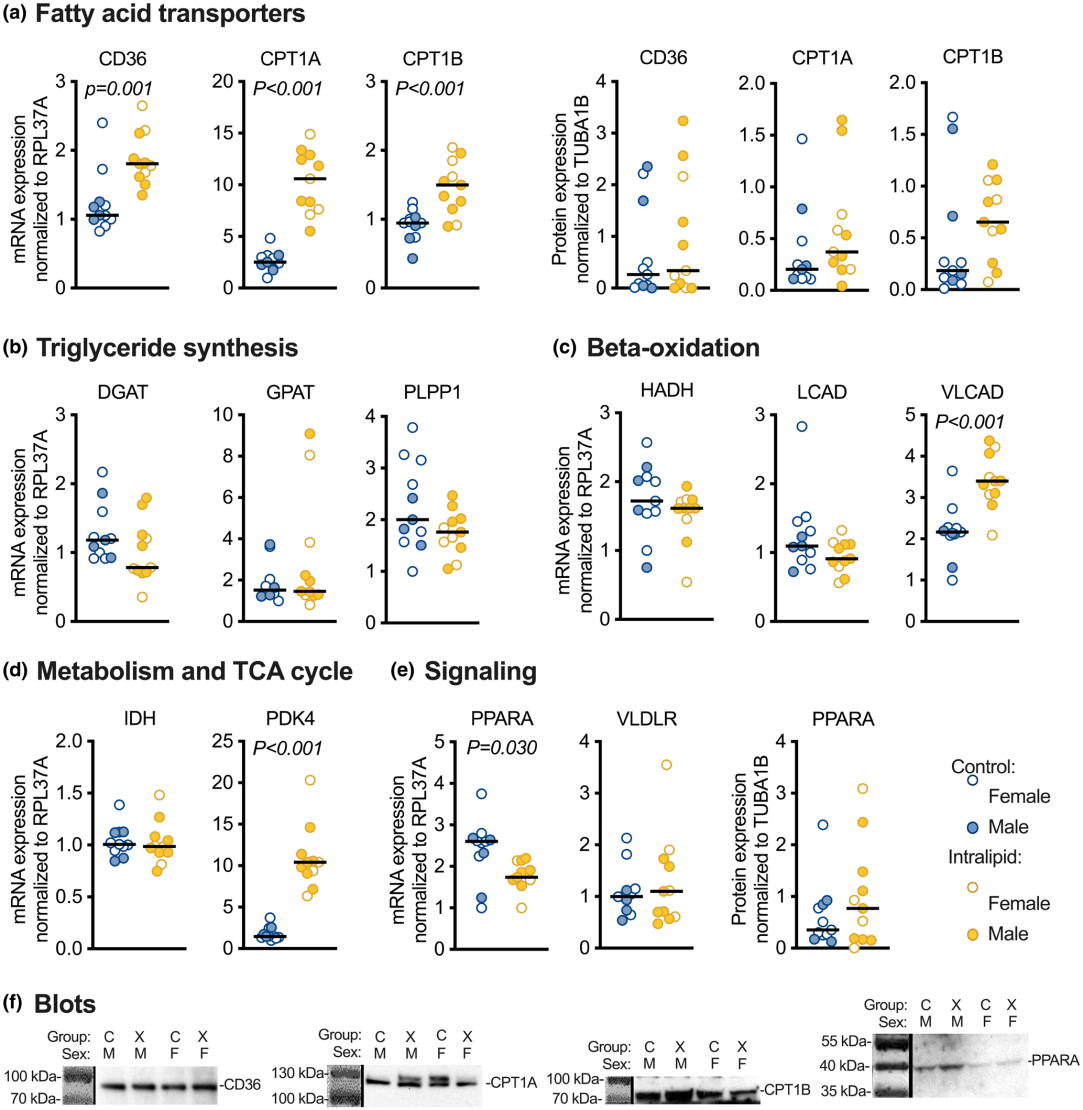
Cardiac expression levels of metabolism‐related genes and proteins following in vivo Intralipid administration in fetal sheep. (a) Fatty acid transporters CD36, CPT1A, and CPT1B mRNA and protein. (b) Esterification and lipid droplet enzymes DGAT, GPAT, and PLPP1 mRNA. (c) Beta‐oxidation enzymes HADH, LCAD, and VLCAD mRNA. (d) Metabolic and TCA cycle enzymes IDH and PDK4 mRNA. (e) Signaling molecules PPARA mRNA and protein, and VLDLR mRNA. (f) Representative images of Western blots. Number for Control female (F) = 7, male (M) = 4; Intralipid female = 4, male = 7. Data were assessed by 2‐way ANOVA. Significant *p* values (*p* < 0.05) shown for differences by treatment; there were no differences by sex.

Intralipid treatment stimulated greater expression of some genes related to fatty acid use and metabolism (Figure 3 and Table 3). Fatty acid transporter and signaling molecule CD36 mRNA was increased by 54% (*p* = 0.001; Figure 3a). CPT1A and CPTA1B catalyze the rate‐limiting transfer of the long‐chain acyl group in acyl‐CoA ester to carnitine, allowing fatty acids to enter the mitochondria for oxidation. CPT1A mRNA was increased by 291% (*p* < 0.001), and CPT1B mRNA was increased by 60% (*p* < 0.001). There were no changes in protein expression for lipid transporters.

Despite increased incorporation of LCFA and VLCFA into intracellular lipid droplets, Intralipid exposure did not change mRNA expression for genes related to esterification and lipid droplet formation (Figure 3b). Of the genes related to beta‐oxidation that were assessed, mRNA expression was 56% greater in Intralipid versus Control for VLCAD (*p* < 0.001; Figure 3c). PDK4 mRNA expression was also increased 500% by Intralipid treatment (*p* < 0.001; Figure 3d). PPARA mRNA was reduced by 26% (Figure 3e; *p* = 0.030) but protein levels were unchanged. PPARA is a nuclear receptor protein functioning as a transcription factor that serves as a lipid sensor and regulates energy consumption and lipid metabolism.

**FIGURE 4:**
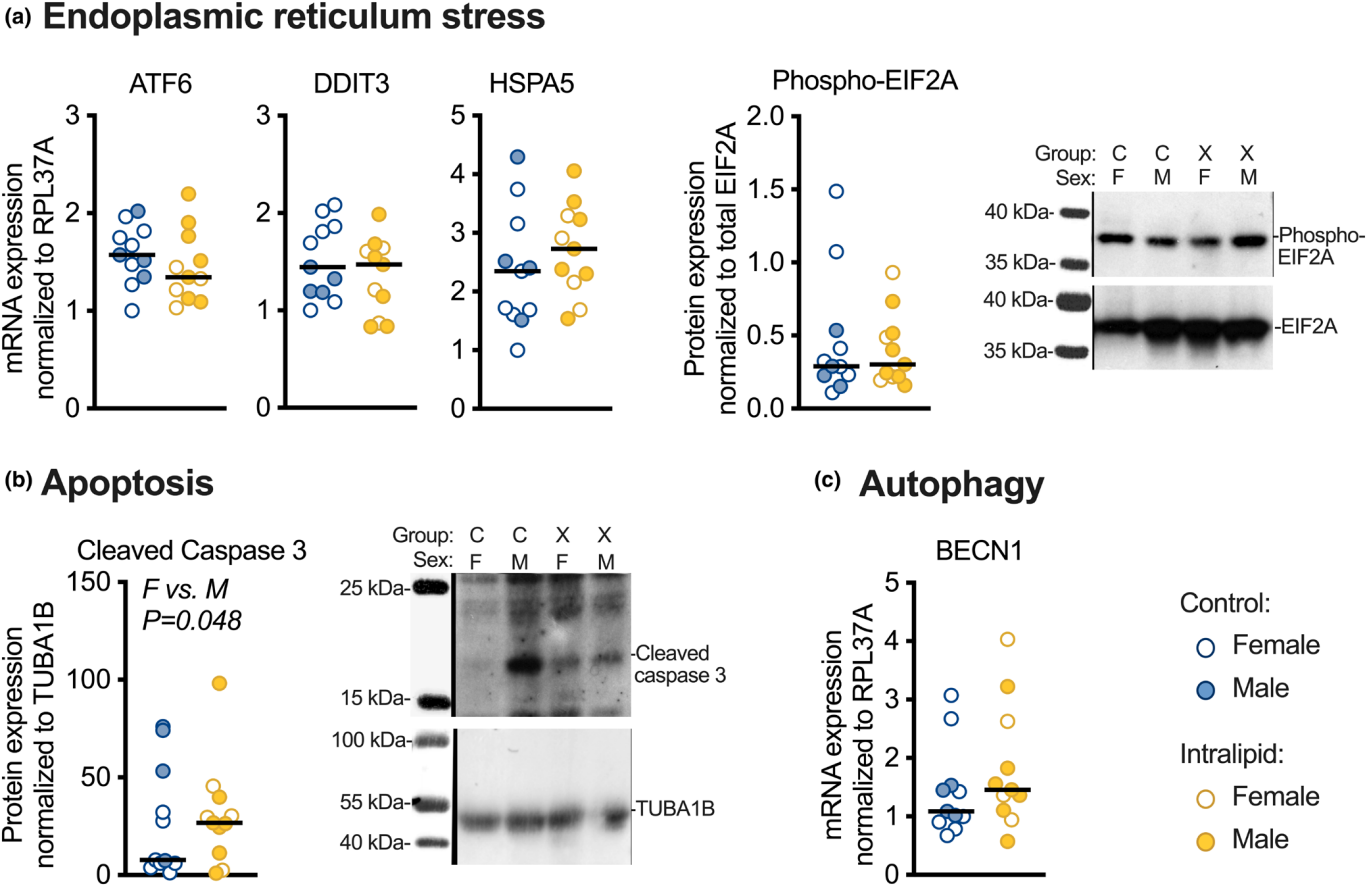
Cardiac expression levels of ER stress, apoptosis, and autophagy‐related genes and proteins following in vivo Intralipid administration in fetal sheep. (a) ATF6, DDIT3 (CHOP), and HSPA5 mRNA, and phospho‐EIF2A protein. (b) Cleaved caspase 3 protein. (c) BECN1 mRNA. Number for Control female (F) = 7, male (M) = 4; Intralipid female = 4, male = 7. Data were assessed by 2‐way ANOVA. Significant *p* values (*p* < 0.05) shown for differences by sex; there were no differences by treatment.

Intralipid treatment did not affect fetal myocardial expression of mRNA or protein related to endoplasmic reticulum stress, apoptosis, or autophagy (Figure 4 and Table 3). Levels of cleaved caspase 3 protein were 56% less in females than in males, regardless of treatment (*p* = 0.048; Figure 4b).

## 4 DISCUSSION

The purpose of this study was to understand how extended exposure to high lipid levels altered the function of late‐gestation fetal cardiomyocytes. As we hypothesized, Intralipid exposure increased the capacity of fetal cardiomyocytes to take up fatty acids. Lipid exposure also changed cardiomyocyte substrate oxidation, although the pattern of regulation was complex. Myocardial gene expression following in vivo Intralipid treatment reflected these changes in lipid handling. Contrary to our hypothesis, ER stress, apoptosis, and autophagy pathways were not activated.

Myocardial mRNA levels of several proteins involved in lipid metabolism appear to be developmentally regulated, including CD36, GPAT, and IDH (Drake et al., 2023). In contrast, mRNA expression of others (for example DGAT, PLPP1, and PDK4) was low during gestation and significantly increased after birth, raising the question of whether this increase was in response to increased circulating lipids due to suckling. In the present study, Intralipid exposure upregulated mRNA levels of CD36, CPT1A and CPT1B, VLCAD, and PDK4, suggesting expression of these genes may be tied to lipid exposure. However, DGAP, PLPP1, and LCAD mRNA levels were not changed by Intralipid exposure, suggesting that their higher expression in newborn lamb hearts is not directly linked to lipid exposure (Drake et al., 2023). Interestingly, in the present study, exposure to Intralipid upregulated fatty acid transporter (CD36 and CPT1) mRNA without changes to protein levels; this mismatch in mRNA and protein expression is also seen in placental insufficiency (Drake et al., 2022).

### 4.1 Cellular respiration

Maximum oxidation of carbohydrates was depressed in cardiomyocytes from Intralipid‐treated compared to vehicle‐treated fetuses, potentially reflecting acceleration towards a mature metabolic phenotype (Lopaschuk & Jaswal, 2010; Razeghi et al., 2001). When cells from these same fetuses were given palmitic acid to oxidize, the difference between the in vivo treatment groups was eliminated, strengthening the interpretation that there was a shift in metabolic substrate preference towards lipids following extended in vivo lipid exposure.

Consistent with these functional changes, fetuses treated in vivo with Intralipid had greater cardiac expression of PDK4 (which increases influx of acetyl‐CoA from beta‐oxidation into the citric acid cycle and slows the rate of glycolysis) and VLCAD (which initiates beta‐oxidation of very long chain fatty acids). Less clear, PPARA mRNA levels were lower in hearts of Intralipid‐treated fetuses, but protein levels were unchanged. PPARA is a lipid‐activated regulator of metabolism and inflammation important in the metabolic switch from carbohydrate to lipid metabolism in the newborn heart, and low PPARA levels in the adult heart are associated with a “fetal‐like” reliance on carbohydrate metabolism and reduced capacity to concentrate triglycerides in the cardiomyocyte (Fillmore et al., 2022; Lefebvre et al., 2006).

Despite extended in vivo lipid exposure potentially maturing fetal cardiomyocyte metabolism, prolonged exposure to increasing levels of lipids in vitro reduced maximal cardiomyocyte respiration in lipid‐containing media. This impairment is reminiscent of the mitochondrial uncoupling observed in cardiomyocyte lipotoxicity (Mthembu et al., 2024; Schulze et al., 2016; Sletten et al., 2018). Consequently, we suggest that both adaptive (maturation) and maladaptive (lipotoxicity) responses are seen when late‐gestation fetal cardiomyocytes are exposed to developmentally inappropriate high lipid levels.

### 4.2 Lipid droplet formation

Myocardial lipid droplets are essential to protect against the lipotoxicity of exposure to acyl intermediates (Barba et al., 2009; Goldberg et al., 2018). They are transient and rise when nonesterified fatty acids in the circulation rise (Goldberg et al., 2018). Long‐term accumulation of lipids within cardiomyocytes is toxic (Schulze et al., 2016; Sletten et al., 2018). Lipid droplet size can determine lipid fate, whether catabolism by lipolysis or lipophagy (Listenberger et al., 2003; Schott et al., 2019), although it is unknown if changes in lipid droplet size in this study cross size ranges important for these processes in the developing heart. While droplets in other tissues can be hundreds of times larger, droplets in cardiac myocytes are typically less than 1 μm in diameter (Wang et al., 2013), as were the diameters in this study (~0.5 μm).

Fetal cardiomyocytes that were exposed to Intralipid in utero took up both long‐chain and very long‐chain fatty acids more rapidly and into bigger lipid droplets than cells from Control fetuses. Previously, we found that hearts of these Intralipid‐exposed fetuses accumulated more intracellular lipids in vivo as assessed by Oil Red O (Piccolo et al., 2024). Thus, extended in vivo exposure to high circulating lipid levels increased the capacity of fetal cardiomyocytes to take up lipids and increased ongoing lipid storage.

Interestingly, despite differences in lipid accumulation, there were no differences in lipid transporter levels or mRNA expression levels of regulators of triglyceride synthesis DGAT, GPAT, and PLPP1. In contrast, CD36 expression, which is indicative of PPAR gamma (PPARG) activity (Bosma et al., 2014), was greater in the hearts of Intralipid‐treated fetuses. PPARG can be protective in cardiac cells exposed to palmitate by upregulating neutral lipid accumulation in lipid droplets but can also contribute to negative effects in long‐term high‐fat diets by increasing cardiomyocyte hypertrophy (Liu et al., 2022). This raises the question of how cardiomyocyte growth is regulated in these Intralipid‐treated hearts at a time of developmental sensitivity to growth modulation (Jonker et al., 2015).

### 4.3 Stress response pathways and cell survival

Palmitate can cause lipotoxicity, leading to apoptosis or necrosis in immature cardiac myocytes (de Vries et al., 1997; Kong & Rabkin, 2000). Cells may react to protect themselves by sequestering lipids, upregulating the unfolded protein response pathways, or initiating autophagy (Palomer et al., 2014; Schott et al., 2019; Yang et al., 2022). Programmed cell death is also a cellular response to protect tissue homeostasis (Dorn 2nd, 2009).

Saturated fatty acids can increase expression levels of ATF6, DDIT3 (also known as CHOP), and HSPA5 (also known as GRP78) mRNA, cause phosphorylation of eEIF2a, and lead to ER stress in cardiac cells (Bosma et al., 2014; Palomer et al., 2014). Interestingly, in our study, mRNA levels of these endoplasmic reticulum stress pathway genes were unchanged, there was no change in phosphorylation of EIF2A, and Intralipid exposure did not activate cleavage of effector caspase 3. High PPARA levels may have suppressed increased expression of endoplasmic reticulum stress pathway genes (Su et al., 2014) and promoted survival (Xu et al., 2020).

PPAR beta/delta (PPARB/D) is activated by fatty acids and can be protective in cardiomyocytes by upregulation of autophagy pathways (Palomer et al., 2014). We assessed Beclin 1, which is regulated by PPARB/D, and found that mRNA expression was unchanged. Unchanged levels of Beclin 1 suggest that autophagy did not contribute to fetal myocyte survival during Intralipid exposure.

### 4.4 Sex differences

Lipid droplet size was greater in female fetuses, and activated caspase 3 levels were lower. These differences may reflect relative protection of immature female hearts in the context of high lipid levels. The hearts of female fetuses have been shown by transcriptomics to be more sensitive to, and perhaps protected from, the maternal lipid environment (Pantaleao et al., 2022).

## 5 CONCLUSION

Although circulating levels are normally low, the fetal myocardium has the capacity to take up and oxidize lipids (Bartelds et al., 2000). Eight days of intravenous lipid infusion at a prenatal age equivalent to mild preterm birth promotes lipid uptake and larger droplet formation in cardiomyocytes, together with upregulation of several key genes. Extended exposure to circulating lipids appears to mature substrate preference in the fetal heart, but any in vitro lipid exposure depresses maximal oxidation capacity. The capacity for lipid metabolism is immature in the late‐term fetal heart, leading to dysfunction, but also adaptation, when exposed to prematurely elevated lipid levels.

## CONFLICT OF INTEREST STATEMENT

The authors declare no conflict of interest.

## ETHICS STATEMENT

All animal experiments were approved by the Institutional Animal Care and Use Committee (#IP0007) and conducted at Oregon Health & Science University.

## Data Availability

Data are provided within the manuscript.
